# Diverse genetic mechanisms underlie worldwide convergent rice feralization

**DOI:** 10.1186/s13059-020-01980-x

**Published:** 2020-03-26

**Authors:** Jie Qiu, Lei Jia, Dongya Wu, Xifang Weng, Lijuan Chen, Jian Sun, Meihong Chen, Lingfeng Mao, Bowen Jiang, Chuyu Ye, Guilherme Menegol Turra, Longbiao Guo, Guoyou Ye, Qian-Hao Zhu, Toshiyuki Imaizumi, Beng-Kah Song, Laura Scarabel, Aldo Merotto, Kenneth M. Olsen, Longjiang Fan

**Affiliations:** 1 Institute of Crop Sciences and Institute of Bioinformatics, College of Agriculture and Biotechnology, Zhejiang University, Hangzhou, 310058 China; 2 Shanghai Key Laboratory of Plant Molecular Sciences, College of Life Sciences, Shanghai Normal University, Shanghai, 200235 China; 3 Rice Research Institute, Yunnan Agricultural University, Kunming, China; 4 Rice Research Institute, Shenyang Agricultural University, Shenyang, China; 5 Department of Crop Sciences, Agricultural School, Federal University of Rio Grande do Sul, Porto Alegre, RS Brazil; 6 State Key Laboratory of Rice Biology, China National Rice Research Institute, Hangzhou, 310006 China; 7 International Rice Research Institute (IRRI), Manila, Philippines; 8 CSIRO Agriculture and Food, GPO Box 1700, Canberra, ACT 2601 Australia; 9 National Agriculture and Food Research Organization (NARO), Tsukuba, Ibaraki, 305-8666 Japan; 10 School of Science, Monash University Malaysia, 46150 Bandar Sunway, Selangor Malaysia; 11 Istituto per la Protezione Sostenibile delle Piante (IPSP), CNR, Viale dell’Università, 16, 35020 Legnaro, PD Italy; 12 Department of Biology, Washington University in St. Louis, St. Louis, MO 63130 USA; 13 James D. Watson Institute of Genome Sciences, Zhejiang University, Hangzhou, 310058 China

**Keywords:** Weedy rice, *Oryza sativa*, Crop feralization, Global population, De-domestication block, Parallel evolution

## Abstract

**Background:**

Worldwide feralization of crop species into agricultural weeds threatens global food security. Weedy rice is a feral form of rice that infests paddies worldwide and aggressively outcompetes cultivated varieties. Despite increasing attention in recent years, a comprehensive understanding of the origins of weedy crop relatives and how a universal feralization process acts at the genomic and molecular level to allow the rapid adaptation to weediness are still yet to be explored.

**Results:**

We use whole-genome sequencing to examine the origin and adaptation of 524 global weedy rice samples representing all major regions of rice cultivation. Weed populations have evolved multiple times from cultivated rice, and a strikingly high proportion of contemporary Asian weed strains can be traced to a few Green Revolution cultivars that were widely grown in the late twentieth century. Latin American weedy rice stands out in having originated through extensive hybridization. Selection scans indicate that most genomic regions underlying weedy adaptations do not overlap with domestication targets of selection, suggesting that feralization occurs largely through changes at loci unrelated to domestication.

**Conclusions:**

This is the first investigation to provide detailed genomic characterizations of weedy rice on a global scale, and the results reveal diverse genetic mechanisms underlying worldwide convergent rice feralization.

**Electronic supplementary material:**

The online version of this article (10.1186/s13059-020-01980-x) contains supplementary material, which is available to authorized users.

## Introduction

Weedy rice (*Oryza sativa* f. *spontanea*) has emerged as a serious global agricultural problem in recent decades [1]. It is distinguished from cultivated rice by a high degree of seed shattering and seed dormancy, by inferior grain quality, and by the ability to aggressively outcompete cultivated rice in paddy fields once established. The evolutionary origin of weedy rice has been debated for decades. Recent evidence from whole-genome resequencing supports origins from cultivated rice ancestors [2–5]. However, genome resequencing investigations to date have been restricted to strains from a single country or region, providing only a limited understanding of the weed’s genomic composition and the timing of weed evolution.

With the recent availability of genome sequence data from > 4500 worldwide cultivated rice varieties [6], it is now possible to infer geographical origins of global weedy rice with a high degree of resolution, and to potentially identify specific varieties that are progenitors of major weed strains. Crop-weed genome comparisons can also provide insights on the genetic mechanisms of weedy rice adaptation. In particular, genome scans can reveal whether adaptations in weedy rice that superficially resemble phenotypic reversions to wild rice (e.g., seed shattering and dormancy) have arisen through changes at the same loci that were targets of selection during domestication (e.g., through second-site suppression of domestication alleles), or through changes at loci that were not involved in the domestication process. Genome sequence analysis can further reveal whether adaptations for weediness in independently evolved strains have occurred through shared or independent genetic mechanisms, thereby shedding light on the extent to which the feralization process is genetically constrained [7, 8].

In this study, we analyzed whole-genome sequences of 524 weedy rice strains, representing all of the world’s major rice-growing regions, together with a worldwide sample of cultivated rice genomes. These analyses reveal that weedy rice has evolved repeatedly from cultivated rice ancestors worldwide, that this process has occurred both early in the history of rice cultivation and very recently, and that despite a high degree of flexibility in the genetic mechanisms by which weediness adaptations evolve, some genomic regions have been targeted repeatedly in the parallel evolution of weed strains.

## Results

### Sampling and genomic data of weedy, cultivated, and wild rice

**Fig. 1:**
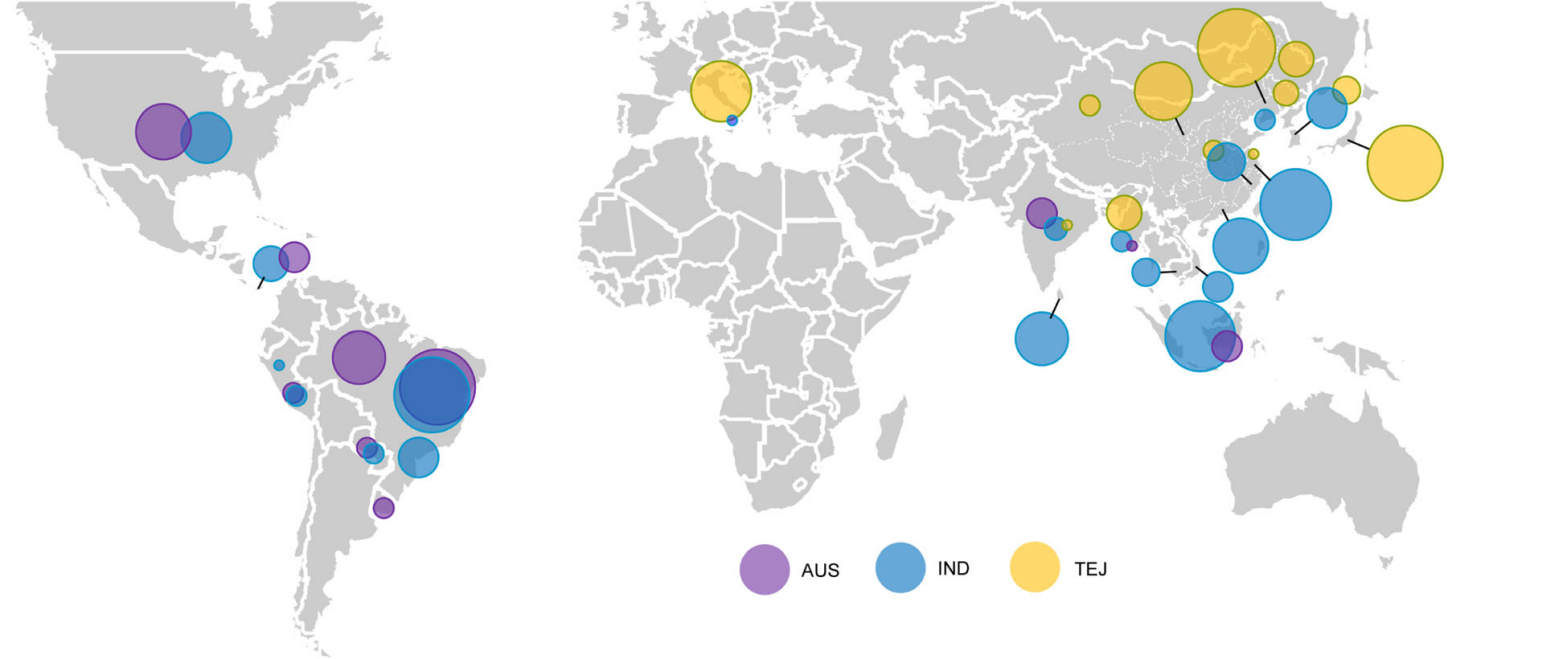
Geographic locations of global weedy rice sampled in this study. Sampling represents 16 countries spanning Asian, European, and American continents. Circle sizes indicate samples sizes by country (from 2 to 50 accessions), and colors indicate inferred crop ancestry (Fig. 2a) (gold for *temperate japonica* (TEJ); blue for *indica* (IND); and purple for *aus* (AUS). Overlapping circles indicate geographic regions with more than one type of weedy rice

For a comprehensive investigation of the origin and evolution of global weedy rice, whole-genome sequences were analyzed from 524 weedy rice accessions representing major rice production areas in 16 countries across Asia, Europe, North America, and Latin America (Fig. 1; Additional file 1: Table S1). Diverse types of rice are cultivated in these regions, including *temperate japonica* (predominant in northern China, Korea, Japan, and Italy), *indica* (in southern China, India, Southeast Asia and Latin America), *aus* (in upland regions of the Indian subcontinent), and *tropical japonica* (in Southeast Asia and the USA). Phenotypically, most of the weed accessions had seeds characterized by reddish-brown pericarp color and smooth spikelet bases, which are typical traits of weedy rice (Additional file 1: Table S1).

Weedy rice samples were sequenced to an average 19.9× genome coverage. For genomic comparison, public genomic sequence data were retrieved for a worldwide sample of 426 locally cultivated rice varieties and 53 wild rice accessions [3, 9, 10]; this yielded a combined genotype dataset of 16.2 million SNPs across 1003 samples for use in the population genomic analysis described below.

### Weedy rice has evolved repeatedly from cultivated rice

**Fig. 2:**
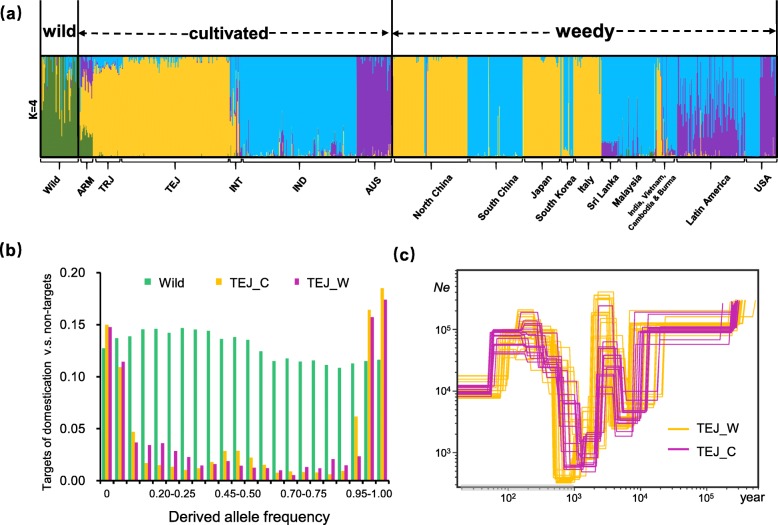
Genetic and geographical origin of global weedy rice. **a** Population structure of global wild, cultivated, and weedy rice. The maximum likelihood clustering with *K* = 4 is presented. The cultivated rice group includes *aromatic*, *tropical japonica*, *temperate japonica*, *indica*, and *aus* type (ARM, TRJ, TEJ, IND, and AUS, respectively). The genetic composition of wild, TEJ, IND, and AUS are colored predominantly with dark green, orange, blue, and purple, respectively. In the weedy rice group, accessions are grouped by country to illustrate the types of weeds in each region; **b** The ratio of allele frequency for SNPs between targets of domestication selection vs. non-targets (estimated by Huang et al. (2012)) for *temperate japonica* type, indicating a similar allele frequency spectrum for *japonica* weedy and cultivated rice. **c** Effective population size (*N*e) changes for the cultivated (C) and different weedy (W) rice populations of *japonica* type. The estimated divergence time between weedy and cultivated rice is shown with the gray-shaded bar along the *x*-axis and assumes one generation per year

Population structure and principal component analysis (PCA) confirmed previously described subgroups within cultivated rice (*tropical japonica*, *temperate japonica*, and *aromatic* varieties within the traditional *japonica* subspecies; and *indica* and *aus* varieties within the traditional *indica* subspecies) (Fig. 2a; Additional file 2: Fig. S1). For weedy rice, though varying by region, all strains shared ancestry predominantly with cultivated rice, specifically varieties of the *indica*, *temperate japonica*, and *aus* subgroups. None of the weeds showed closest ancestry with wild rice, although some degree of wild rice introgression was evident in Southeast Asian strains based on FastStructure and ABBA analyses (Fig. 2a; Additional file 2: Fig. S2); this is consistent with previous inferences of wild rice introgression into weed populations in this geographical region [11–13]. Assessments of genome-wide nucleotide diversity indicated that most weedy groups harbor lower genetic diversity than their respective inferred crop ancestors (Additional file 2: Fig. S3), consistent with post-domestication bottlenecks during feralization. We also compared the ratio of the derived allele frequency spectrum of genomic regions that were targets of selection during rice domestication [9] and regions that were not, in wild, cultivated and weedy rice populations. Both *japonica* and *indica* weeds showed the domestication-associated U-shaped distribution found in cultivated rice (Fig. 2b; Additional file 2: Fig. S4) and thus bear a signature of ancestry from domesticated ancestors. Similarly, the relative genetic diversity change of domestication and improvement genes [14] shows a similar level of reduction for weedy and cultivated rice compared to wild rice (Additional file 2: Fig. S5); this is again consistent with weedy rice descent from domesticated ancestors.

Estimates of divergence times between weeds and their respective crop ancestors revealed substantial variation among strains (Fig. 2c; Additional file 2: Fig. S6). For example, both *japonica-*derived weeds and cultivated *japonica* rice shared a very recent genetic bottleneck around 1000 years ago (assuming one generation per year) (Fig. 2c), and had similar patterns in the distribution of population effective size (*N*_e_) after that; this suggests that *japonica* weeds likely diverged from their cultivated counterparts < 1000 years ago. In comparison, some *indica*- and *aus*-derived weeds were inferred to have diverged from their respective crop ancestors approximately a millennium earlier (Additional file 2: Fig. S6). Taken together, these results suggest that weedy rice has evolved repeatedly and independently from cultivated ancestors at different time points during the history of rice cultivation.

### Hybrid origin of Latin American weedy rice

Weedy rice in Latin America (e.g., Brazil, Panama, Paraguay, and Peru) is unique among worldwide samples, with over half of samples (51/95) showing admixed genetic ancestry between *indica* and *aus* (Fig. 2a; Additional file 1: Table S1, S2). Consistent with this pattern, a TreeMix analysis indicates that these putatively admixed accessions originated from hybridization between Latin America *indica-* and *aus-*type weedy rice (Additional file 2: Fig. S7). In addition, the admixed strains showed elevated nucleotide diversity compared to local *indica* or *aus* weedy rice strains (Additional file 2: Fig. S3), as well as higher observed heterozygosity than weedy rice in other world regions (Additional file 2: Fig. S8). Given that weedy rice, like cultivated rice, is predominantly self-fertilizing, these patterns suggest that many of the Latin American weeds in our sample have originated through recent hybridization of local *aus* and *indica* weeds.

Commercial varieties with ALS (acetolactate synthase) herbicide resistance (HR) have been released in many countries since 2001, and several weedy rice populations with tolerance to herbicide have been reported recently [15–17]. In our weedy rice collection, a total of 11 non-synonymous SNPs were found within ALS in 52 Latin American weedy rice accessions, mostly from Brazil (51, including 9 *indica* type, 4 *aus* type, and 38 *indica-aus* type) (Additional file 1: Table S3; Additional file 2: Fig. S9a). Most HR cultivars in Brazil are *indica*. Of the 11 ALS mutations observed in Latin American weeds, 3 functional mutations (Ala122Thr, Ser653Asn, and Gly654Glu) have been employed in HR cultivars [18]. These results suggest that HR weedy rice has likely acquired resistance by crop-weed hybridization and adaptive introgression—i.e., escape of resistance alleles from HR cultivars—although the possibility of parallel HR evolution in weedy rice by mutational convergence cannot be ruled out with the present data.

### Convergent in situ origins of weedy rice from local cultivars

Kinship analysis was carried out to assess geographical origins of weedy rice from each sampled region or country. With the exception of the USA, where weed strains were likely introduced from Asia [2, 19], most weedy rice worldwide appears to have originated from local cultivars or varieties grown in neighboring regions (Additional file 2: Fig. S10; Additional file 1: Table S4). For example, weed accessions from southern China (Jiangsu, Guangdong, and Zhejiang) were inferred to be closest to Chinese cultivated varieties; over half of weeds from northern China (Liaoning and Jilin) have highest kinship with the cultivars from the nearby Korean peninsula; and Japanese weeds show high kinship with cultivars from South Korea and Japan. Most weeds in Southeast and South Asia also show close relationships with cultivars from local or neighboring countries. In a parallel pattern, Italian weedy rice was inferred to be most closely related to European cultivars.

**Fig. 3:**
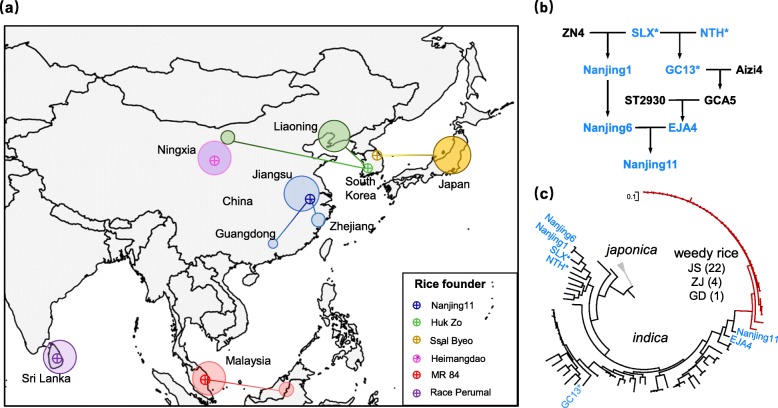
Rice cultivar founders for multiple Asian weedy rice populations. Based on kinship analysis, six rice cultivars are inferred to have given rise to multiple weedy rice strains in the regions where the cultivars are/were grown. Circle sizes indicate sample sizes (from 1 to 25 accessions per circle). **b** The pedigree of Nanjing11. The accessions which are genome re-sequenced in this study are colored in blue, and the pre-Green Revolution cultivars (non-dwarf) are indicated by an asterisk “*”. NTH: Nantehao; SLX: Shenglixian; GC13: Guangchang13; Aizi4: Aizhaizhan4; GCA5; Guangchangai5; ST2930: Situo2930; ZN4: Zhongnong4; NJ1: Nanjing1; EJA4: Erjiuai4. **c** Phylogenetic tree for the Nanjing11 pedigree accessions and weedy rice which have the highest kinship with Nanjing11. The branch of weedy rice including 22, 4, and 1 sample from Jiangsu (JS), Zhejiang (ZJ) and Guangdong (GD), China, is indicated in red. In addition to six Nanjing11 pedigree accessions, a total of 50 other *indica* cultivars which are the closest to the weedy rice group were also included in the tree. Three *japonica* accessions, in gray, are used as an outgroup

Notably, the kinship analysis further revealed multiple cases where individual formerly widely-grown cultivars have apparently given rise to the major contemporary weed strains in the region where the cultivar was once grown (Additional file 1: Table S5; examples shown in Fig. 3). For example, a total of 38 weeds from Liaoning in northern China showed highest kinship with a single widely grown twentieth century cultivar “Huk Zo” while 16 Japanese weeds showed highest kinship with “Ssal Byeo.” Both Huk Zo and Ssal Byeo are Korean landraces. For Malaysia, one variety “MR 84,” which was released in 1986 and widely planted during 1980s to 1990s, has a total of 12 weedy rice accessions with closest kinship relationship. In China, one cultivar, “Nanjing11,” was found to be closest to 27 weed strains from South China (Jiangsu, Zhejiang, and Guangdong). This cultivar was bred around 40 years ago in Nanjing, Jiangsu Province; it remained one of the most popular *indica* cultivars and was broadly cultivated throughout South China until about 15 years ago, when it was replaced by newer cultivars. These patterns suggest that a large proportion of Asian weed strains are descended from commercial cultivars that were widely grown in the twentieth century, as rice agriculture shifted from smallholder farms to industrialized production.

To further document this pattern, we collected parental pedigree accessions of Nanjing11 and re-sequenced their genomes (Fig. 3b). The phylogenetic tree confirmed the group of 27 weed strains has closer kinship with Nanjing11 than its pedigree accessions (e.g., EJA4), and the topology supported that the weedy rice group is likely to be derived from Nanjing11, not its parental lines before Green Revolution (e.g., GC13, SLX, and NTH) (Fig. 3c). Extrapolating from these results, we can estimate that more than 35% (27/75) of the current weedy rice strains in southern China (Jiangsu, Zhejiang, Guangdong) are likely descended from Green Revolution cultivars. Taken together, these results indicate that widely grown twentieth century cultivars that were developed during the Green Revolution have left a legacy of weedy rice infestations throughout Asia.

### Non-domestication genomic regions for adaptation of weedy rice

**Fig. 4:**
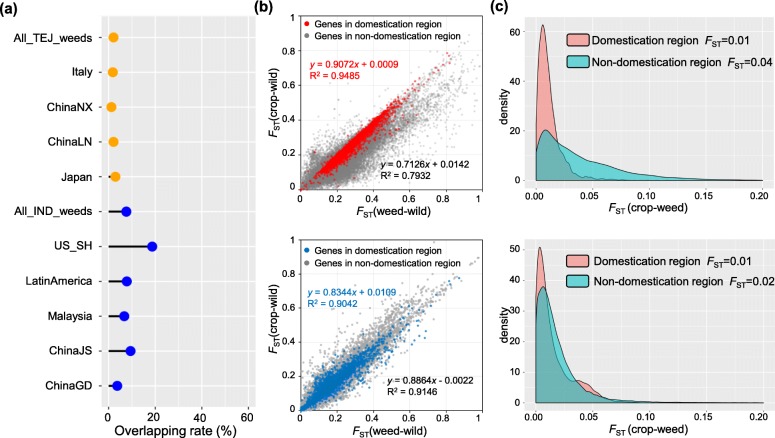
Comparison between weedy and cultivated rice in domestication and non-domestication regions. **a** The overlapping rate (%) between de-domestication regions of weedy rice and domestication regions of rice. The *japonica-* and *indica-* type weedy rice groups are indicated by orange and blue, respectively. ChinaJS and ChinaGD refer to weeds from the Chinese provinces of Jiangsu and Guangdong, respectively. **b** The genes in targeted genomic regions of domestication selection are highlighted in red for *japonica* type (top), and blue for *indica* type (bottom), while gray points indicate genes locating outside of domestication regions. The *x*-axis represents *F*_ST_ value between weedy and wild rice populations, and *y*-axis represents *F*_ST_ value between cultivated and wild rice populations. **c** The distribution for *F*_ST_ values of each gene between weedy and cultivated rice is plotted, with red distribution showing genes residing in domestication region and green distribution for non-domestication genes

To identify genomic regions with signatures of adaptive differentiation between weed strains and their inferred cultivated ancestors, genomic scans of differentiation were performed (Z(*F*_ST_) > 3) (Additional file 2: Fig. S11). We further examined whether these significantly differentiated regions between weedy and cultivated rice overlap with domestication regions. Notably, a very low overlapping rate was observed for most weeds worldwide (the exceptions being regions of South and Southeast Asia where wild rice hybridization has led to adaptive introgression of wild alleles at domestication loci [11–13, 20]). An especially low rate, 2.1% (1.2–2.9%), was observed for *japonica* type weeds, while for *indica* type, the mean overlapping rate (excluding South and Southeast Asia) was 7.6% (3.7–18.7%) (Fig. 4a; Additional file 1: Table S6). In addition, we found that genes in regions not known to be associated with domestication show higher differentiation between weedy and cultivated rice, particularly for *japonica* type weeds (Fig. 4b, c); this indicates that after diverging from cultivated rice, natural selection may act more strongly on the genomic regions unrelated to domestication loci.

Consistent with this pattern of adaptive divergence, we found many mutations in weedy rice that were not observed in cultivated varieties (Additional file 2: Fig. S12). Novel variation in the *ALS* gene for herbicide resistance provides one such example. Among the 11 non-synonymous *ALS* SNPs identified in weed strains (described above), a mutation (Ala205Val) has not been previously reported in cultivated, wild, or weedy rice (Additional file 1: Table S3). An herbicide resistance assessment revealed that the weed accessions with this ALS mutation showed strong herbicide (Imazamox) resistance (Additional file 2: Fig. S9b). The presence of this mutation suggests that weed populations can evolve resistance through new spontaneous mutations.

### De-domestication blocks under parallel evolution

**Fig. 5:**
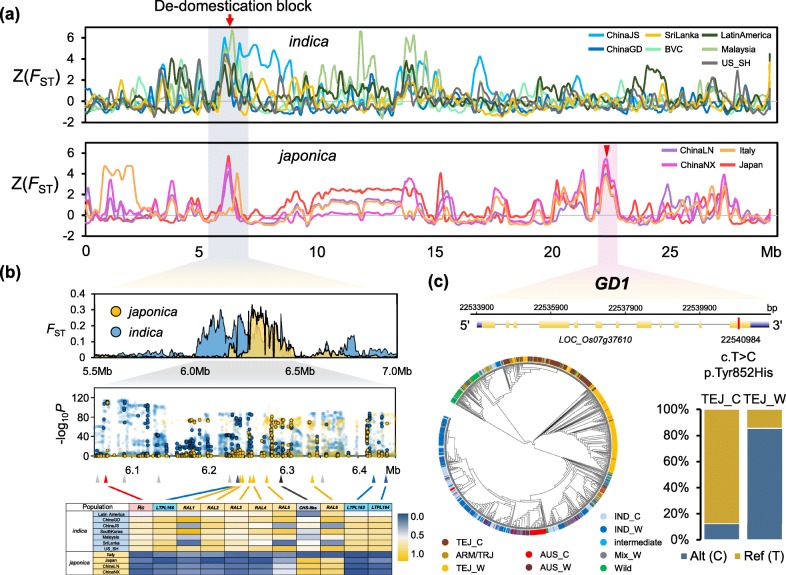
Genomic differentiation and parallel selection in global weedy rice. **a** Normalized genomic differentiation (*F*_ST_) of different groups of *indica* and *japonica* weedy rice compared with its related type of cultivated rice for chromosome 7. Two novel loci were labeled using arrows. **b** A common divergent genomic region in weedy rice genome. Above, *F*_ST_ scan of the genomic region from 5.0 to 7.0 Mb in chromosome 7. Middle, significance of the allele frequency difference in the region. Bottom, the extent of divergence (blue: low; yellow: high) for genes within the region including *Rc*, six *RAL*s, three *LtpL*s and one *CHS*. **c** Characterization of a seed germination-related gene *GD1*, which is under parallel selection in *japonica* weeds. The phylogeny of the gene shows clear separation of TEJ cultivars and weeds. One non-synonymous SNP (T to C in weedy population) at the 12th exon causes an amino acid change and significantly high frequency of the variant was observed in weedy population

Despite the independent and repeated origins for different weedy rice populations, we can find some shared genomic regions that are highly diverged from cultivated rice, indicating that these regions may underlie shared targets of selection in weed evolution (we refer to these as de-domestication “hot blocks,” to distinguish them from more localized hotspots). One of the most significantly differentiated regions is a 0.5-Mb de-domestication hot block from 6.0 to 6.5 Mb on Chromosome 7 in both *japonica-* and *indica*-type weeds (Fig. 5a). This region harbors multiple genes (e.g., *Rc*, *RAL*, and *LtpL*) with potential functions for environmental adaptation (Fig. 5b). For example, *Rc* pleiotropically controls both red pericarp and seed dormancy [21]. The red pigment in rice grains is caused by proanthocyanidins or condensed tannins, which could have deterrent effects on pathogens and predators [22]. Seed dormancy is a highly adaptive trait for weedy rice, as it enhances survival of seeds in the soil seed bank and allows seeds to persist in rice fields over multiple seasons [23]. Interestingly, a cluster of six *RAL* (seed allergenic protein) genes and three *LtpL* genes (encoding plant lipid transfer proteins that function in alpha amylase inhibition) are also located within this region. All nine genes harbor the protein domain PF00234 (Protease inhibitor/seed storage/LTP family). These genes are proposed to be involved in multiple roles, such as inhibiting the growth of fungal and bacterial pathogens and facilitating adaptation of plants to various environmental conditions [24], which may protect the weedy rice seeds from pathogens and predators in paddy fields for years during their dormancy. For *indica*-type weeds, each of the *RAL*s showed clear differentiation from those in their cultivated ancestors (*F*_ST_ > 0.4) (Additional file 2: Fig. S13). Our results thus suggest that the *RAL* genomic region has been a repeated target of selection in the evolution of weed strains from cultivated rice.

Another de-domestication hot block occurs in the 22.5–23.1-Mb genomic region of chromosome 7, which stands out as the highest peak when comparing all combined *japonica* weedy rice with cultivars, and also with a *Z*-value of ~ 4 for each *japonica*-type weedy rice population compared to *japonica* cultivated rice (Fig. 5a; Additional file 2: Fig. S11). Within this region resides a gene encoding B3 domain containing transcription factor *GD1*, which participates in regulating GA and carbohydrate homeostasis, and further regulates rice seed germination and seedling development [25]. The phylogeny of *GD1* clearly shows that most *japonica* weedy rice strains are separated from the group of *japonica* cultivated rice, which is consistent with the haplotype of this gene (Fig. 5c). In addition, we found one non-synonymous SNP on the last exon, and the allele frequency is markedly different between *japonica* weedy (0.85) and cultivated (0.12) rice. The results above suggest that the seed germination-related gene is under potential parallel evolution among different *japonica* weedy rice populations and may play a crucial role for the distinct germination behavior of weedy rice compared to cultivated rice in rice fields.

## Discussion

In this study, we have found that de-domestication is a primary mechanism for the origin of weedy rice globally, and most weeds are derived from a single genetic founder population (*indica*, *japonica*, or *aus*). In particular, some weedy rice populations can be traced to a single local contemporary cultivar that has apparently served as the founder of the local weedy rice population. Historically, a high diversity of varieties has been cultivated on a local scale in Asia [26–28]. The proliferation of modern Asian weed strains has followed widespread shifts during the Green Revolution towards industrialized rice farming and adoption of a few high-yielding elite cultivars, which our data show to be direct founders of many contemporary Asian weed strains. Whether there are some special traits and underlying genetic basis in those candidate weed-derived founders is an interesting question for further investigation. The results will provide valuable practical implications for crop breeding and weed control.

We have also observed that the majority of the de-domestication-related loci fall outside the regions which have been targets of selection during domestication. In other words, it appears that regions unrelated to selection during domestication were differentially targeted during the de-domestication process. Interestingly, the finding is more obvious for the recently evolved *japonica* type weedy rice group than *indica* group. A possible explanation could be due to relatively higher diversity of the *indica* group, which would harbor more standing genetic variation that could facilitate adaptation during weed evolution.

Although weedy rice originated independently and repeatedly throughout the rice domestication history, we found genomic evidence for shared targets of selection in different weed populations worldwide (Fig. 5a). Just as Li et al. [2] suggested that weediness of weedy rice can emerge through selection on “genomic islands,” we believe that this “genomic island strategy” could provide weedy rice with high fitness and a rapid adaptive ability, requiring few generations and minimal genomic recombination for weed evolution.

## Conclusions

Based on the largest global weedy rice panel to date, we found weedy rice has evolved multiple times independently from domesticated ancestors, and most Old World strains have in situ origins within the regions where they presently occur. Despite the inbreeding nature of cultivated rice, large-scale *indica-aus* hybridization accounts for the origin many Latin American weed strains. Some of the most aggressive contemporary Asian weed strains appear to have very recent origins from now-discontinued Green Revolution elite cultivars. Genome scans suggest that feralization occurs largely through changes at loci that were not targets of selection during domestication. Moreover, independently evolved weed strains have some shared “de-domestication” genomic blocks (including regions containing candidate genes for seed dormancy and allergenic proteins), indicating that there are shared genomic targets of selection in parallel feralization events.

## Methods

### Plant sampling and DNA sequencing

A total of 524 weedy rice samples were collected from 16 countries worldwide where the rice fields are heavily infested by weedy rice. New whole-genome sequence data were generated for 327 weedy rice accessions from nine Asian countries (China, South Korea, Japan, Malaysia, Burma, Vietnam and Cambodia, Sri Lanka, and India), one European country (Italy) and five Latin American countries (Brazil, Panama, Paraguay, Peru, and Uruguay). Paired-end sequence data were generated by Illumina HiSeq4000 and Illumina X Ten. In addition, our previous genome resequencing data, including 197 accessions of weedy rice from the USA and China [2, 3], were also integrated. The average genomic coverage for the 524 weedy accessions is 19.9×. Other genomic data from 479 *Oryza* accessions were obtained from a previous study [9, 10]. These included 159 *indica* varieties; 146 *temperate japonica* varieties; 41 *tropical japonica* varieties; 17 *aromatic* varieties; 49 *aus* varieties; 14 rice varieties of intermediate classification; and 53 wild *O. rufipogon* accessions (Additional file 1: Table S1).

### Variant detection and genotyping

The raw paired-end reads were first filtered into clean data using NGSQCtoolkit v2.3.3 [29]. The cut-off value for PHRED quality score was set to 20 and the percentage of read length that met the given quality was 70. Clean reads of each accession were mapped to rice reference genome (MSUv6.1) using BOWTIE2 v2.2.1 [30] with default settings. Consecutive steps using Samtools v0.1.19 [31] and GATK v3.7 [32] were applied for variant detection. Potential PCR duplicates were removed by “Samtools rmdup.” Alignments around small indels were remapped with “ndelRealigner,” and raw variants were called based on the realigned bam file. Using the called variants as known sites, “BaseRecalibrator” and “PrintReads” in the GATK were applied for base-pair score recalibration. The resulting BAM files of each sample were used for the multi-sample variant genotyping. “UnifiedGenotyper” in GATK was applied to generate the raw variant calls with parameters “-stand_call_conf 30, -stand_emit_conf 10”. To reduce the variants false discovery rate, the SNP calls were filtered according to the following threshold: QUAL < 30, QD < 2, MQ < 30, MQ0/DP > 0.1. Potential variant annotation and effect were predicted by SnpEff v3.6 [33]. Imputation was performed by BEAGLE v4.0 [34] using the genotype likelihoods and specified the number of iterations to 10.

### Population structure and gene flow analysis

Based on the genome-wide SNPs among the 1003 wild, cultivated, and weedy rice accessions, principal component analysis was performed by SNPRelate v0.9.19 [35]. FastStructure [36] was applied to infer the ancestry of the 1003 rice accessions with *K* values ranging from 3 to 8. The phylogenetic tree was constructed using Fasttree [37] with 1000 bootstrap replicates.

### Derived allele frequency comparison for domestication and non-domestication regions

The rice domestication regions for *japonica* and *indica* rice were retrieved from Huang et al. [9] and re-positioned from the genome reference of IRGSP Build 4 to MSUv6.1. The allele frequency of domestication-related and domestication-unrelated regions was calculated for wild, cultivated, and weedy rice, respectively. Genomic data of 10 *O. barthii* samples from Wang et al. [38] were used for genotyping of the SNPs identified in our 1003 rice panel. The genotype of *O. barthii,* which was determined by at least 8 individuals, was used to polarize SNPs as either ancestral or derived. Finally, the ratio of derived allele frequency between domestication-related and domestication-unrelated regions was calculated.

### Origin inference by kinship analysis

Based on the 5.23 million SNP dataset of 4591 cultivated rice which was collected and imputed by Wang et al. [6], we implemented kinship analysis to infer the origin of each weedy rice sample. As the genome reference of Wang et al. [6] was based on MSUv7, we first transformed the 5.23 million SNP sites from MSUv7 to MSUv6.1. The 150-bp sequence up- and downstream of each SNP site was extracted and aligned to rice genome MSUv6.1 by blast, and transformed the sites by in-house PERL script. We genotyped these sites for all 524 weedy rice using GATKv3.7 [32]. Kinship was calculated with TASSEL5 using Centered IBD method [39]. Based on kinship matrix, we obtained cultivars with highest kinship for each weedy rice sample. Then, we determined the geographical origin of each weedy rice origin based on the location of cultivars.

### Identification of genomic regions under selection

The genome was scanned in a 100-kb window size with a step size of 10 kb, and the population parameters (*π*, *F*_ST_) were estimated for each window by VCFtools [40]. In the genomic differentiation analyses based on population differentiation index (*F*_ST_), we compared weedy rice of one group against the totality of its potential ancestors (e.g., Italian weeds vs. all temperate *japonica* cultivars). *Z*-transformation was applied to locate divergent regions between weedy rice and cultivated rice from the extreme tails by applying a threshold of three standard deviations.

### Demography inference

The divergence time between cultivated rice and weedy rice of *japonica*, *indica*, and *aus* type was inferred using SMC++ v1.13 [41], which could estimate the effective population size history based on whole-genome sequence data and is powerful for recovering history for short time scales. Three lineages in each population, respectively, were randomly selected as the distinguished individuals to improve the estimation of effective population size. The divergence time range was estimated according to the distinct change of effective size between cultivated and weedy populations. The mutation rate was assumed as *μ* = 6.5 × 10^− 9^ mutations × bp^− 1^ × generation^− 1^ [42], and 1 year per generation were adopted.

### Introgression analysis

To examine the possible introgression from wild rice into Malaysian weedy rice, ABBA-BABA statistics (also known as “Patterson’s D” or “D statistics”) were implemented. ABBA-BABA statistics tested for admixture between three closely related populations “P1,” “P2,” and “P3,” using an outgroup population “O” following the phylogeny (((P1, P2), P3), O). In our analysis, *Oryza barthii* was used as outgroup (O), while Malaysian *indica*, weedy rice, and wild rice were served as P1, P2, and P3, respectively. Genotyping data were transferred to “geno” format using parseVCF.py within genome_general (https://github.com/simonhmartin/genomics_general). For genome-wide D statistics, genotyping frequency was first calculated with freq.py employed in genome_general. Then D was calculated by custom R scripts following the equation from Green et al. [43]. The TreeMix v1.13 software [44] was also adopted to explore the potential gene flow between different groups of weedy or cultivated rice.

### GO enrichment analysis

GO enrichment analysis was carried out using AgriGO with “*Oryza sativa* MSU6.1 nonTE” set as species background (http://bioinfo.cau.edu.cn/agriGO/) [45]. The *P* value and false discovery rate (FDR) (Yekutieli) criteria of < 0.0001 and < 0.05, respectively, were used for the considered enrichment GO terms.

### Imazamox resistance assessment

Clean seeds were sown in plastic boxes (24 cm × 30 cm × 9 cm) containing 60% silty loam soil, 15% sand, 15% perlite, and 10% peat by volume and placed in a greenhouse for germination. A population that has never been treated with herbicide was used as reference susceptible population (10–1). After 15 days, the seedlings were thinned to 20 per box and two replicates of 15–20 plants for each population were treated with Imazamox at the recommended field rate of 35 g a.i. ha^−1^ (Altorex, BASF, 40 g a.i L^−1^, solution). The herbicide was applied on seedlings at the 3–4 leaf stage, using a bench sprayer equipped with three flat-fan (extended range) hydraulic nozzles (Teejet, 11002) delivering 300 L ha^−1^ at a pressure of 215 kPa. Four weeks after treatment, the number of surviving plants was assessed. Plants were considered dead if, regardless of color, they showed no active growth. Standard error (SE) was calculated for each mean.

## Supplementary information

**Additional file 1: Table S1.** Information of all rice accessions used in this study. **Table S2.** Genetic subgroup assignments of global weedy rice samples used in this study. **Table S3.** Distribution of ALS non-synonymous mutations in wild, cultivar and weedy rice. **Table S3.** Kinship relationship for each weedy rice sample with cultivated rice in the panel including 4591 rice accessions. **Table S5.** The candidate ‘hub’ cultivated rice that has high number of weedy rice accessions with highest kinship. **Table S6.** The overlapping rate between de-domestication regions of weedy rice with rice domestication regions.

Additional file 2: Figure S1. Principal component analysis (PCA) plot of 1003 rice accessions including weedy, cultivated and wild rice by the first and second eigenvectors. **Figure S2.** Distribution of D-statistic (±s.e.) across 12 chromosomes for Malaysian weedy rice. **Figure S3.** Genetic diversity (*π*) of whole-genome and domestication-related genes in wild, cultivar and weedy rice populations. **Figure S4.** The ratio of allele frequency for SNPs targets of domestication selection versus non-targets for *indica* type. **Figure S5.** Comparisons of genetic diversity for domestication and improvement genes between weedy and cultivated rice. **Figure S6.** Effective population size change of *indica* and *aus* cultivated and weedy rice. **Figure S7.** Phylogenetic relationship of different weedy rice groups and possible introgression inferred from TreeMix analysis. **Figure S8.** Heterozygosity level evaluated by observed heterozygosity across wild, cultivated and weedy rice populations. **Figure S9.** Weedy rice with ALS-inhibiting herbicide resistance. **Figure S10.** Geographic origins of weedy rice traced by Kinship analysis. **Figure S11.** Genomic differentiation regions for different weedy populations compared to their counterparts in cultivated rice. **Figure S12.** Numbers of SNPs that are shared or unique among weedy and cultivated rice. **Figure S13.** Characterization of one of the genes (*RAL6*) encoding allergenic proteins in the de-domestication genomic block.

Additional file 3 Review history.
