# Supplementary material for: Diverse genetic mechanisms underlie worldwide convergent rice feralization
Source: Genome Biol. 2020 Mar 26;21:70. doi: 10.1186/s13059-020-01980-x (PMC7098168; doi:10.1186/s13059-020-01980-x)
Supplement: Supplementary file 2 — Principal component analysis (PCA) plot of 1003 rice accessions including weedy, cultivated and wild rice by the first and second eigenvectors. Figure S2. Distribution of D-statistic (±s.e.) across 12 chromosomes for Malaysian weedy rice. Figure S3. Genetic diversity (π) of whole-genome and domestication-related genes in wild, cultivar and weedy rice populations. Figure S4. The ratio of allele frequency for SNPs targets of domestication selection versus non-targets for indica type. Figure S5. Comparisons of genetic diversity for domestication and improvement genes between weedy and cultivated rice. Figure S6. Effective population size change of indica and aus cultivated and weedy rice. Figure S7. Phylogenetic relationship of different weedy rice groups and possible introgression inferred from TreeMix analysis. Figure S8. Heterozygosity level evaluated by observed heterozygosity across wild, cultivated and weedy rice populations. Figure S9. Weedy rice with ALS-inhibiting herbicide resistance. Figure S10. Geographic origins of weedy rice traced by Kinship analysis. Figure S11. Genomic differentiation regions for different weedy populations compared to their counterparts in cultivated rice. Figure S12. Numbers of SNPs that are shared or unique among weedy and cultivated rice. Figure S13. Characterization of one of the genes (RAL6) encoding allergenic proteins in the de-domestication genomic block. [file 13059_2020_1980_MOESM2_ESM.pdf]

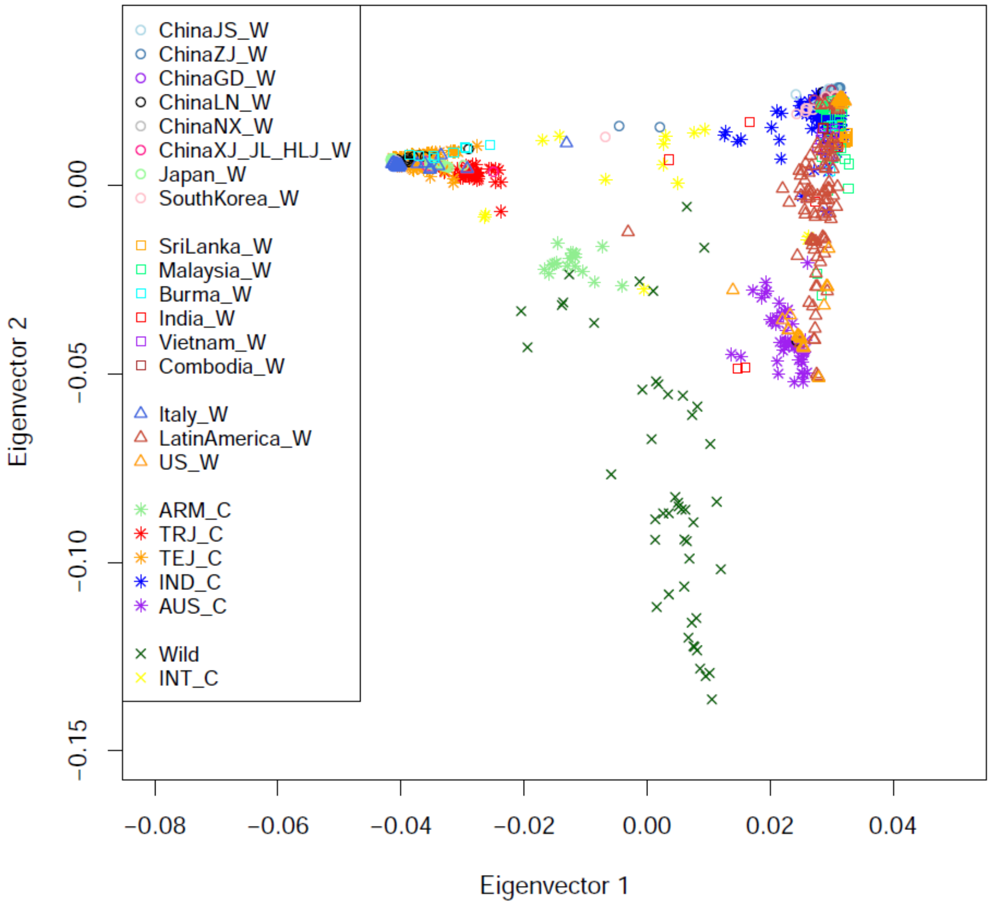

Fig S1. Principal component analysis (PCA) plot of 1003 rice accessions including weedy, cultivated and wild rice by the first and second eigenvectors. Weedy rice accessions from East Asia are labeled with open circles ('○') while weeds from South or Southeast Asia labeled with open squares ('□'). Weedy rice from Italy, North America and Latin America are indicated with open triangles ('△'). Cultivated rice is indicated by asterisks ('*'), and wild type rice by 'x'. Abbreviations for each province in China are as follows: JS: Jiangsu; ZJ: Zhejiang; GD: Guangdong; LN: Liaoning; NX: Ningxia, XJ: Xinjiang; JL: Jiling; HLJ: Heilongjiang. BVC stands for Burma, Vietnam and Combodia.

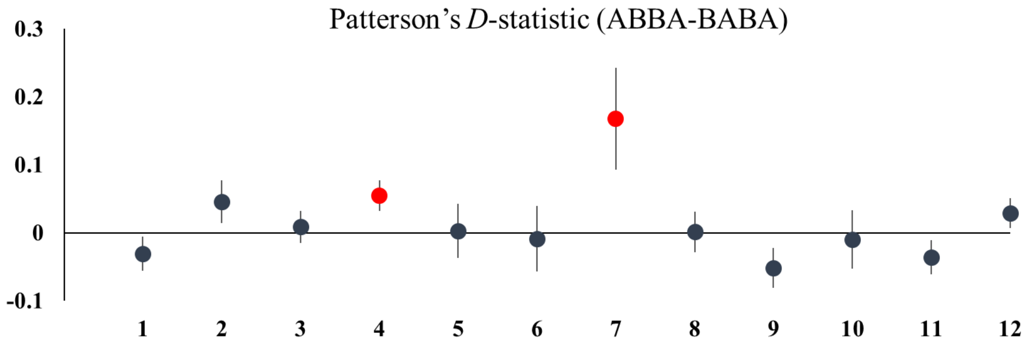

Fig S2. Distribution of D-statistic (±s.e.) across 12 chromosomes for Malaysian weedy rice. Chromosomes indicated with red dots are those with significant deviations from the null expectation (0) for the test of ABBA excess (from wild to weedy rice) relative to BABA (from wild to cultivated rice).

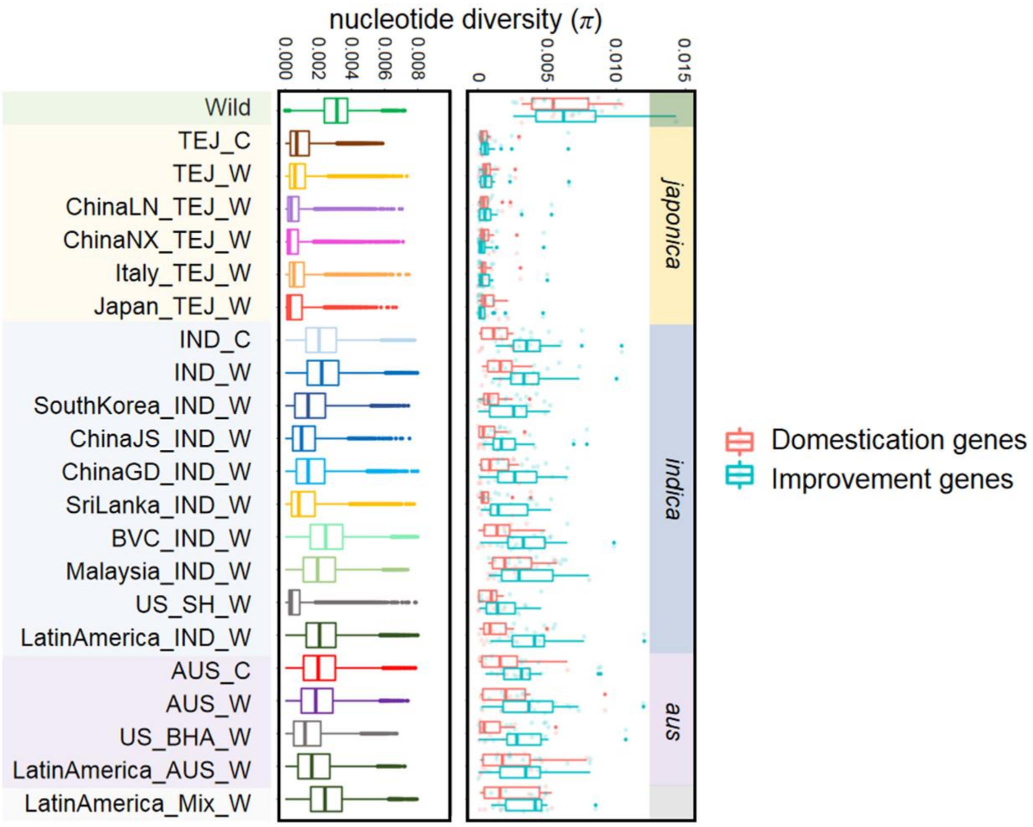

Fig S3. Nucleotide diversity (π) of whole-genome and domestication-related genes in wild, cultivar and weedy rice populations.

Abbreviations for each province in China are as follows: JS: Jiangsu; ZJ: Zhejiang; GD: Guangdong; LN: Liaoning; NX: Ningxia, XJ: Xinjiang; JL: Jiling; HLJ: Heilongjiang. In addition, BVC stands for Burma, Vietnam and Cambodia. 'TEJ', 'IND', 'AUS' stand for temperate *japonica*, *indica*, and *aus* type, respectively. The suffix 'C' and 'W' refer to cultivated and weedy rice, respectively. The 'LatinAmerica_Mix_W' stands for one Latin American weedy rice group with *indica-aus* mixed genetic composition. The domestication-related gene list was retrieved from Chen et al. (2019).

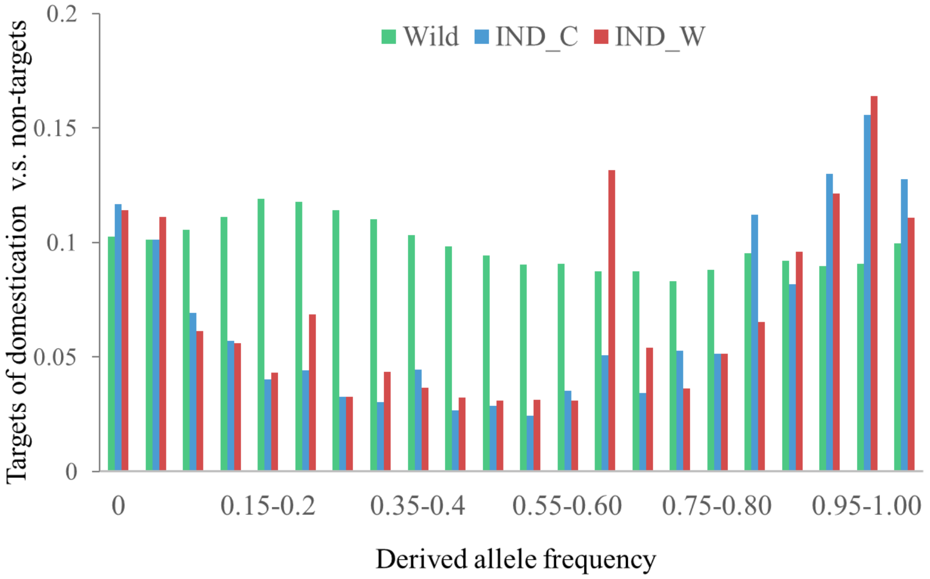

Fig S4. The ratio of allele frequency for SNPs targets of domestication selection versus non-targets for *indica* type.

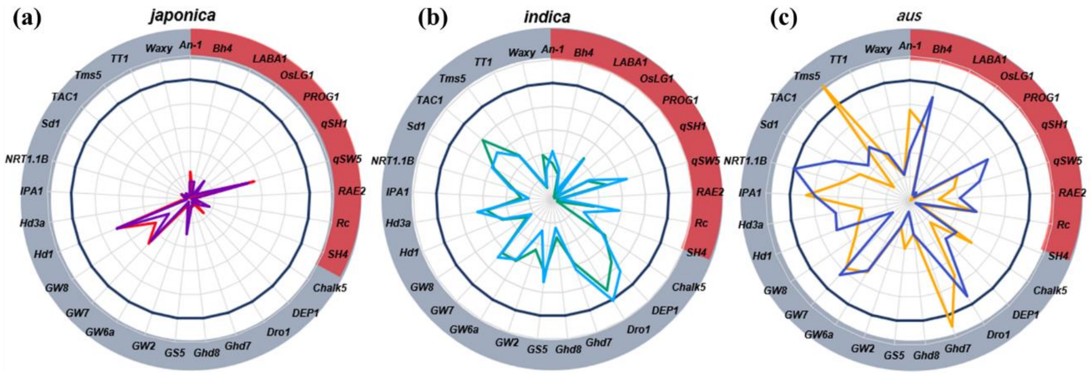

Fig S5. Comparisons of genetic diversity for domestication and improvement genes between weedy and cultivated rice. Genes in the red background represent domestication genes and those in the gray background represent improvement genes. Red and purple lines indicate *japonica* cultivars and weedy rice, respectively (**a**); green and light blue lines indicate *indica* cultivars and weedy rice, respectively (**b**); and yellow and dark blue lines indicate *aus* cultivars and weedy rice, respectively (**c**). π values are normalized compared to diversity of wild rice (indicated by black lines).

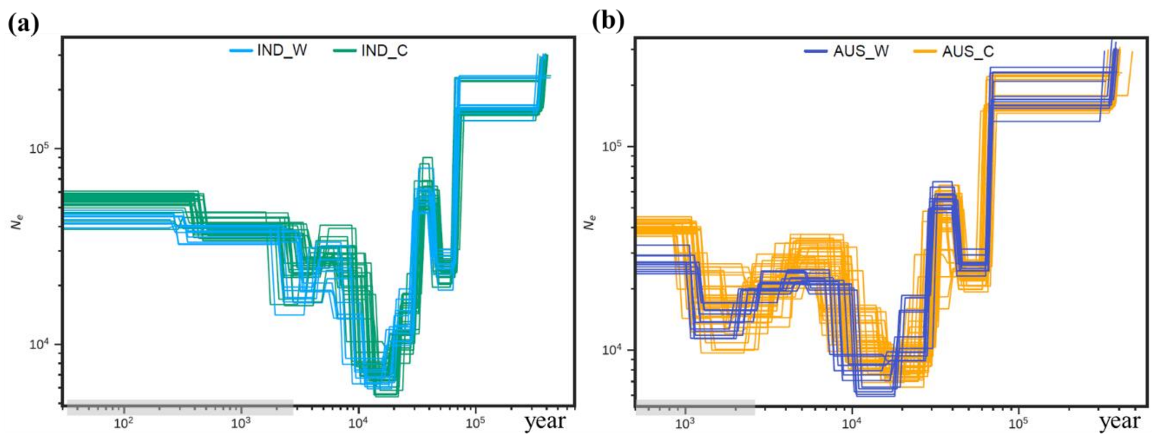

Fig S6. Effective population size change of *indica* (**a**) and *aus* (**b)** cultivated rice and their weedy derivatives. Estimated divergence time (generations ago) between cultivated and weedy rice is marked with gray shaded bars along *x* axis.

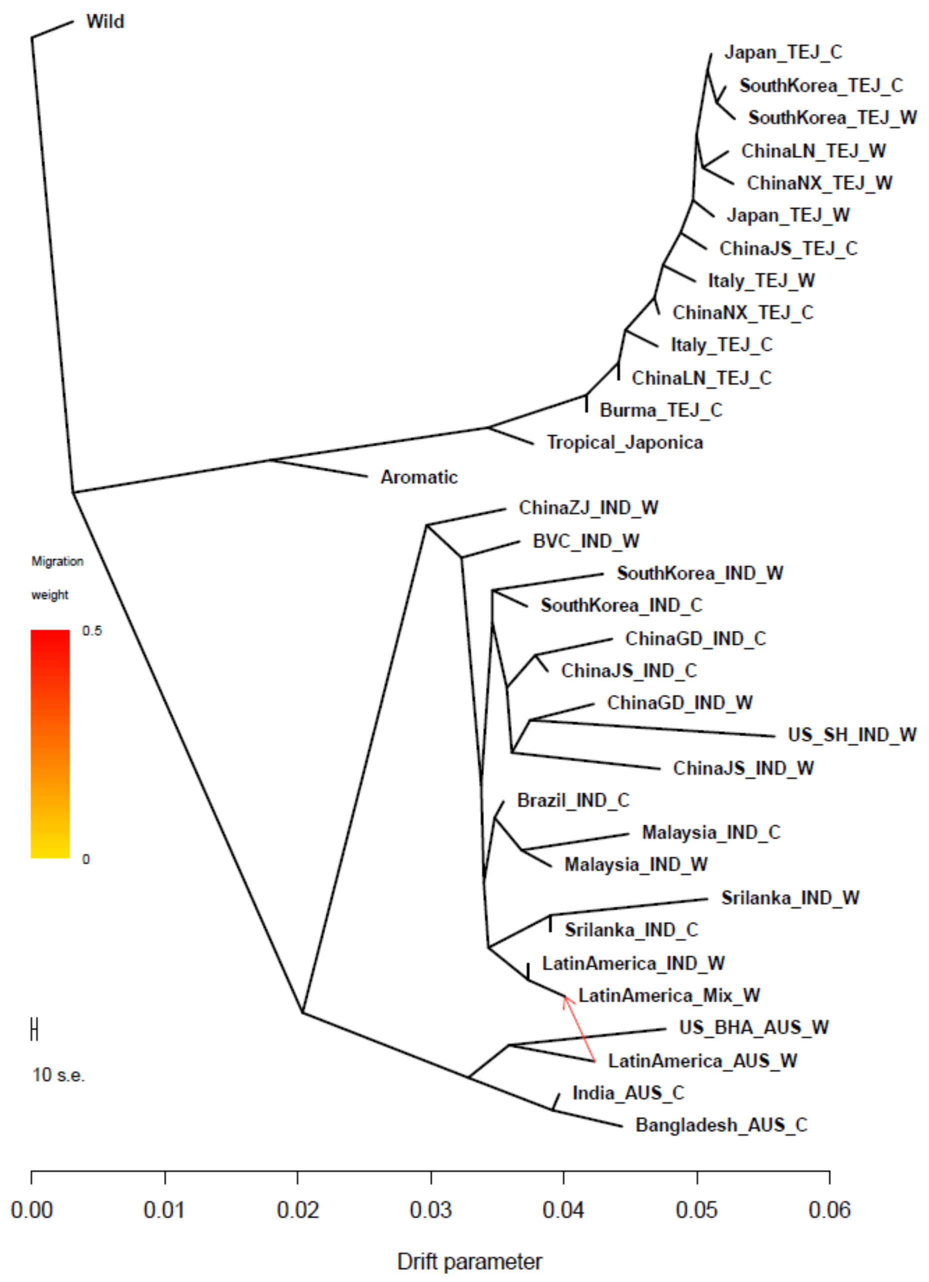

Fig S7. Phylogenetic relationship of different weedy rice groups and possible introgression inferred from TreeMix analysis. Arrows indicate the direction of gene flow. while the line colors represent the migration weight based on sample number.

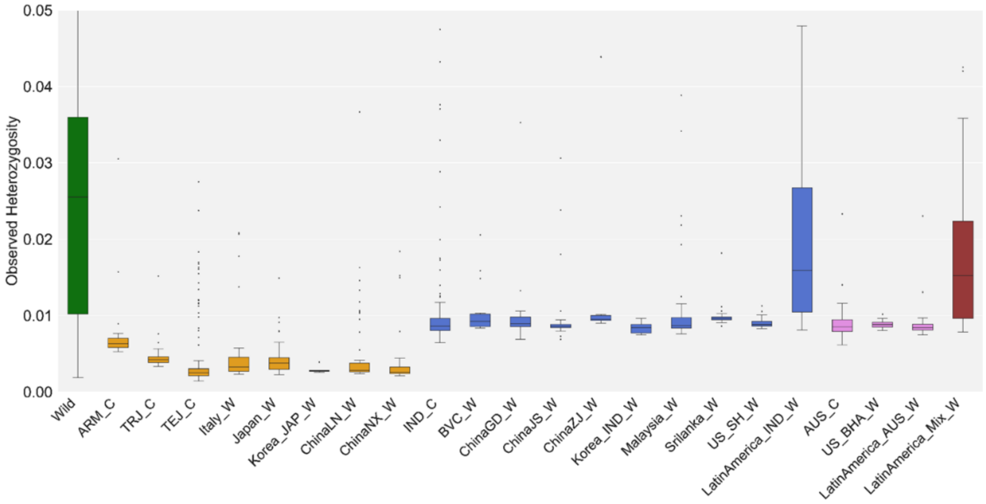

Fig S8. Heterozygosity level evaluated by observed heterozygosity across wild, cultivated and weedy rice populations.

(a)

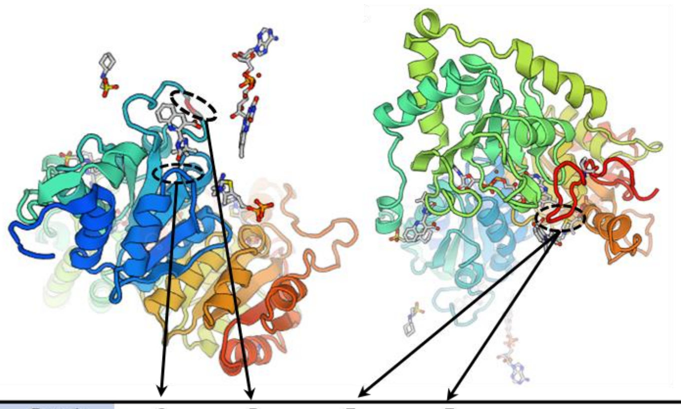

Mutation	Domain	C	D	E	E		
	Position	Ala122Thr	Ala205Val	Ser653Asn	Gly654Glu	Mutated samples	Total samples
Region	Brazil	25 (5)*	0	4 (1)	24 (7)	51	84
	Paraguay	1	0	0	0	1	1
	Panama	0	1	0	0	1	7
	Italy	0	1	6	0	7	38
	Total	26	2	10	24	60	130

(b)

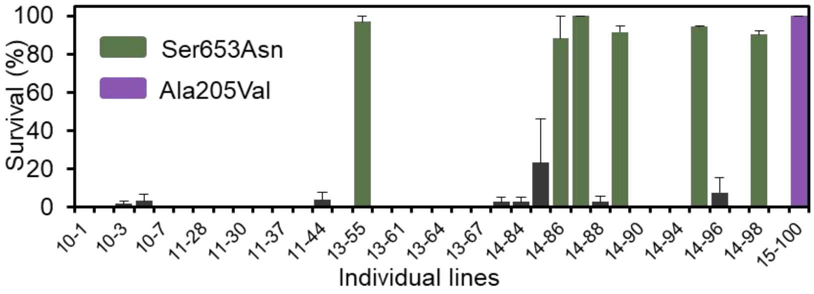

Fig S9. Weedy rice with ALS-inhibiting herbicide resistance. (a) Distribution of herbicide-resistance causative mutations in geographical regions. The number in the bracket indicate the number of weedy rice accessions with heterozygous alleles. (b) Examination of survival percentage of Italian weedy accessions with mutations in ALS gene. Green bars represent samples with Ser653Asn mutation, purple bars indicate Ala205Val mutation, and the grey bars indicate samples with no mutations found.

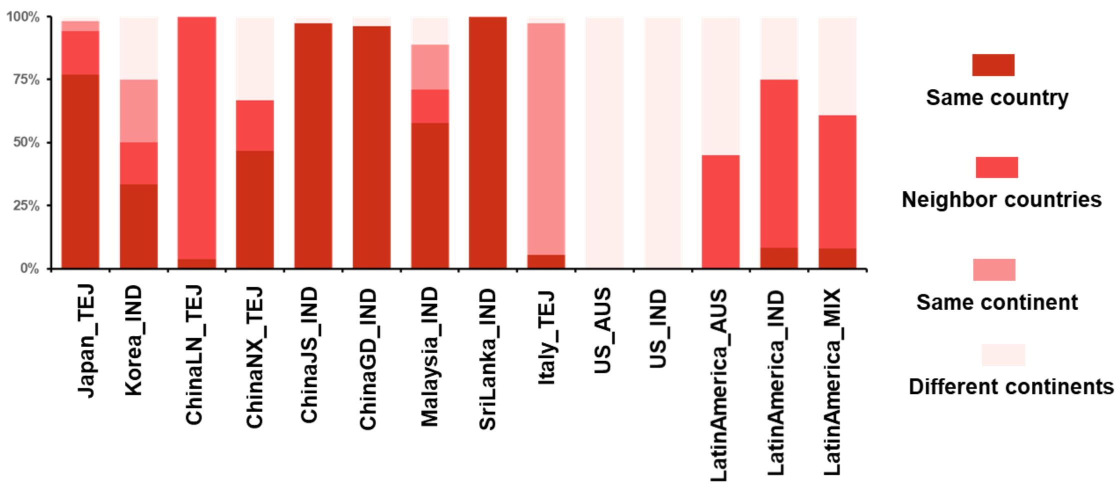

Fig S10. Geographic origins of weedy rice traced by Kinship analysis. Four levels of geographic relationships are colored from dark to light (i.e. Same country, neighboring country, same continent and different continent). Abbreviations for each province in China are as follows: JS: Jiangsu; GD: Guangdong; LN: Liaoning; NX: Ningxia. 'TEJ', 'IND', 'AUS' stand for *temperate japonica*, *indica*, and *aus* type, respectively. LatinAmerica_MIX group stands for one Latin American weedy rice group with *indica-aus* mixed genetic composition.

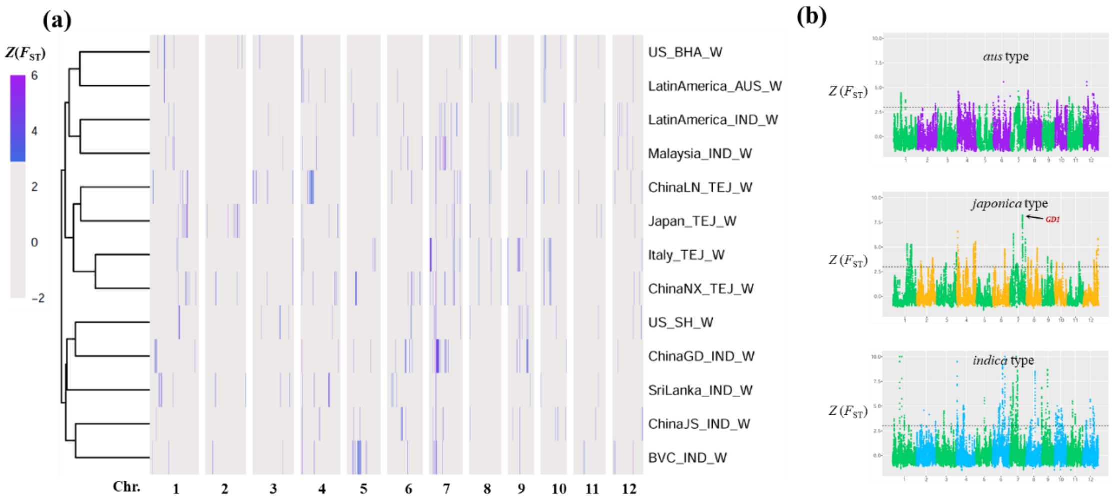

Fig S11. Genomic differentiation regions for different weedy populations compared to their counterparts in cultivated rice. (a) Significant differentiated genomic windows ($Z(F_{ST}) > 3$) for each weedy group are highlighted in blue and purple. Clustering is based on the Z values of each comparison. (b) The weedy rice accessions of the same type are grouped and compared to their counterparts in cultivated rice.

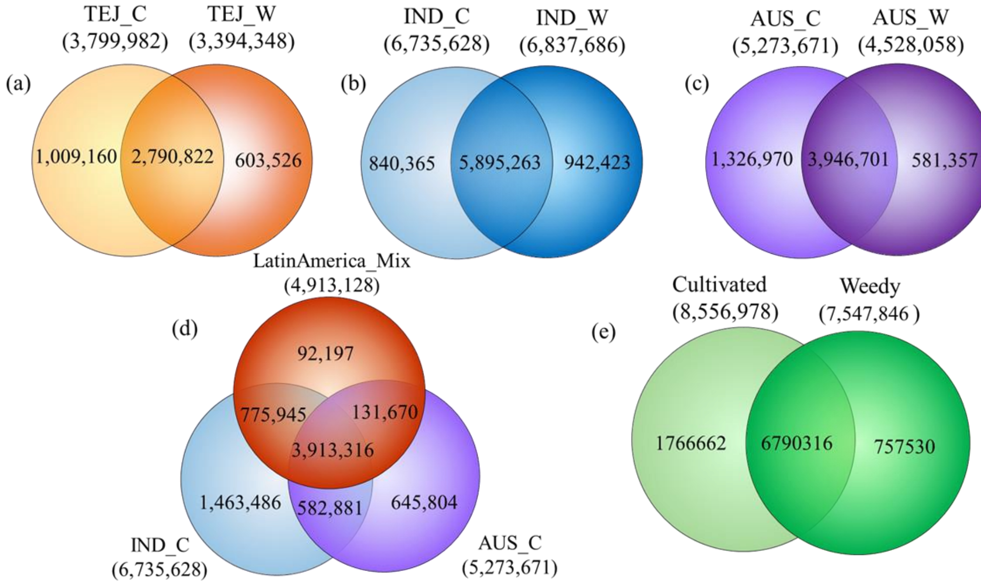

Fig S12. Numbers of SNPs that are shared or unique among weedy and cultivated rice. The different groups are color-coded as follows: light blue, light orange, violet for *indica*, *japonica* and *aus* cultivated rice, respectively; The weedy rice populations are color-coded as follows: Japonica type: dark orange (a), indica type: dark blue (b), aus type: purple (c), Latin American indica-aus mixed type: brown (d); All cultivated rice and all weedy rice are color-coded as light green and green, respectively (e).

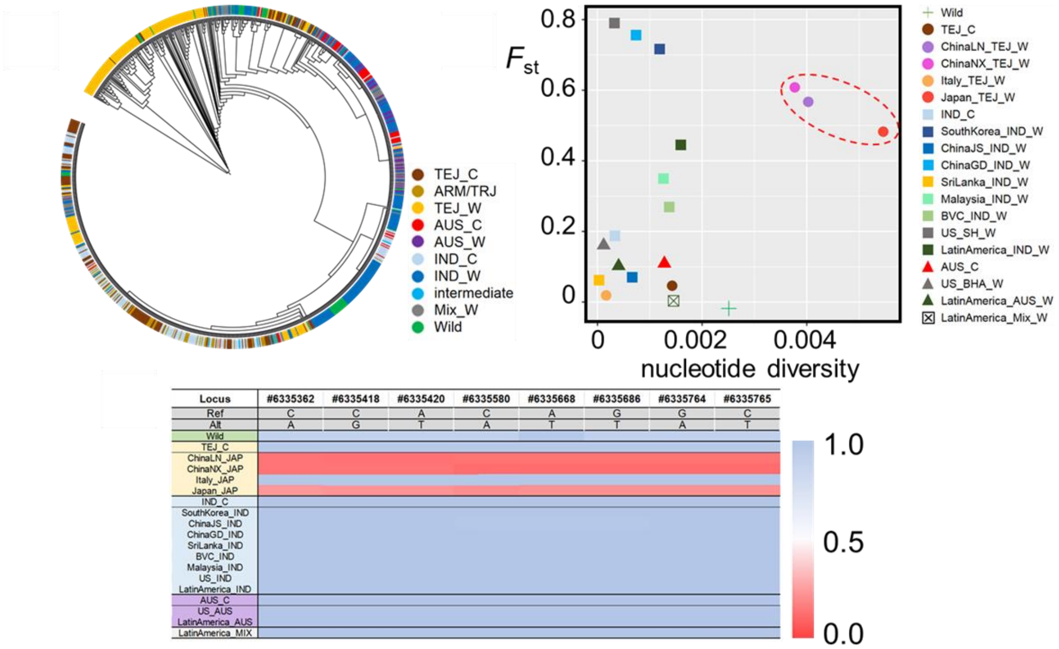

Fig S13. Characterization of one of the genes (*RAL6*) coding allergenic proteins in the de-domestication genomic block. (**a**) Phylogenetic tree of *RAL6*. (**b**) Nucleotide diversity and F_{ST} of *RAL6* in different populations. For weedy populations, F_{ST} values represent differentiation between weedy rice and cultivated rice, while for cultivated rice, F_{ST} represent differentiation between cultivated and wild rice. Three outliers of *japonica* weedy population were highlighted by red dashed circle. (**c**) Allele frequency distribution of 8 missense mutations in *RAL6*. Reference-type allele frequency was shown from 0 (red) to 1 (blue). Colored icons represent different populations as legends in (b) show.
