# Supplementary material for: Diverse genetic mechanisms underlie worldwide convergent rice feralization
Source: Genome Biol. 2020 Mar 26;21:70. doi: 10.1186/s13059-020-01980-x (PMC7098168; doi:10.1186/s13059-020-01980-x)
Supplement: Supplementary file 3 — Review history. [file 13059_2020_1980_MOESM3_ESM.docx]

Review History

**First round of review**

**Reviewer 1**

**Are you able to assess all statistics in the manuscript, including the appropriateness of statistical tests used?** No, I do not feel adequately qualified to assess the statistics.

**Comments to author:**

This paper describes a comprehensive, worldwide survey of genomic variation in weedy rice.  The authors show that weedy rice generally evolves locally from local rice varieties, with some weak introgression from wild rice.  The exception are Latin American weedy rices, which appear to be admixed. Selection analysis shows that genomic regions under selection in weedy rice differ from those seen in domestication, suggesting different targets of selection.

In general this is an interesting paper and the analysis is good.  The sampling is also unprecedented give the number of samples and the wide geographic scope of the sampling.  The analyses are through and comprehensive, and overall this is a good addition to the literature.  I do not find any major issues with the analyses or conclusions.

**Reviewer 2**

**Are you able to assess all statistics in the manuscript, including the appropriateness of statistical tests used?** Yes, and I have assessed the statistics in my report.

**Comments to author:**

This manuscripts describes the origin and evolution of a worldwide collection of weedy rice accessions. The extensive sampling, deep sequencing, and clear genomic comparisons address questions of how the conspecific weed is related to wild and cultivated rice lineages, how the evolution of weediness relates to the genetic basis of domestication, and how similar the genetic basis of weediness is across the broad samples of weedy accessions. Overall, the data is solid, the manuscript is generally well-written, and the work will provide the basis for a great deal of future research in addition to answering the standing questions listed above. Having read the manuscript several times, I did have some questions that the authors can answer through relatively minor revisions and have listed these below. I look forward to seeing the revised manuscript in press in the future.

**Key words:** Consider adding “Oryza sativa” to your key words, as it does not occur in your title or abstract.

**Methods:**

- L320-324: What sequencing platform and chemistry did you use to generate these sequences? This does not seem to be mentioned in the manuscript or in the supplementary files.

- L362-371, Derived allele frequency methods/results: It’s not clear to me why you used the ratio of the derived allele frequency in domesticated vs. undomesticated regions. What is the utility of this compared to a standard site frequency spectrum? This seems like a needless layer of complexity to add to the paper, and makes it difficult to interpret Figure 2b. I do expect a domesticated species to show a U-shaped distribution of derived allele frequencies, but I have no similar expectation for a ratio of allele frequencies.

- L426-437 – What populations are these? Does this include all the weedy rice accessions?

**Results:**

- ALS herbicide resistance: In L169-173 you state that because some of the herbicide resistance mutations found in domesticated rice are also present in weedy rice this indicates introgression of the alleles from the crop to the weed. While this is the most parsimonious explanation, you have not eliminated the other possibility, which is parallel mutation through mutations at the same sites. This is not uncommon in the evolution of herbicide resistance, and must be addressed in the current manuscript.

- L267: I can’t quite understand the meaning of “in the same cluster and separate with” as you describe the phylogeny here.

- L 269: Did you mean “is different” instead of “is differed”?

**Discussion:**

- L277-279: Here, you say that “most weeds are derived from a single genetic background”. I assume you mean that most are not hybrids, but it sounds like you are saying that most are derived from the same founder population. I would suggest revising.

- General suggestions for the origin of weeds from green revolution cultivars: I thought that this was perhaps the most interesting finding of the paper. However, I didn’t realize how many of the 524 weedy accessions had been derived from these lines until I looked at TableS5 – I believe the total is 269 (unless some weeds are double-counted)? If the total is really this high, please add it to the body of the paper – it will make a much more impressive impact if the reader can see that over 50% of weeds are derived from GR lines. Also, I think it might be pertinent to mention that this was a time when farming methods advanced considerably around the world. It is possible that a shift in farming methods contributed to the coordinated rise of these weeds.

**Authors Response**

**Point-by-point responses to the reviewers’ comments:**

*Reviewer reports:

Reviewer #1: This paper describes a comprehensive, worldwide survey of genomic variation in weedy rice.  The authors show that weedy rice generally evolves locally from local rice varieties, with some weak introgression from wild rice.  The exception are Latin American weedy rices, which appear to be admixed. Selection analysis shows that genomic regions under selection in weedy rice differ from those seen in domestication, suggesting different targets of selection.

In general this is an interesting paper and the analysis is good.  The sampling is also unprecedented give the number of samples and the wide geographic scope of the sampling.  The analyses are through and comprehensive, and overall this is a good addition to the literature.  I do not find any major issues with the analyses or conclusions.*
**Response:** Your positive comments about the quality of the paper are highly appreciated. We believe this work will provide a useful contribution to the literature, and will be valuable for further investigations by other researchers in this field in the future.

*Reviewer #2: This manuscripts describes the origin and evolution of a worldwide collection of weedy rice accessions. The extensive sampling, deep sequencing, and clear genomic comparisons address questions of how the conspecific weed is related to wild and cultivated rice lineages, how the evolution of weediness relates to the genetic basis of domestication, and how similar the genetic basis of weediness is across the broad samples of weedy accessions. Overall, the data is solid, the manuscript is generally well-written, and the work will provide the basis for a great deal of future research in addition to answering the standing questions listed above. Having read the manuscript several times, I did have some questions that the authors can answer through relatively minor revisions and have listed these below. I look forward to seeing the revised manuscript in press in the future.* 
**Response:** We greatly appreciate your positive comments. We hope this global weedy rice work will have a lasting impact in the literature in future.

*Key words: Consider adding “**Oryza sativa” to your key words, as it does not occur in your title or abstract.*
**Response:** “*Oryza sativa*” has been added as one of the key words.

*Methods: 
- L320-324: What sequencing platform and chemistry did you use to generate these sequences? This does not seem to be mentioned in the manuscript or in the supplementary files.*
**Response:** We have added the information into the Methods section. “Paired-end sequence data were generated by Illumina HiSeq4000 and Illumina X Ten.”

*- L362-371, Derived allele frequency methods/results: It’s not clear to me why you used the ratio of the derived allele frequency in domesticated vs. undomesticated regions. What is the utility of this compared to a standard site frequency spectrum? This seems like a needless layer of complexity to add to the paper, and makes it difficult to interpret Figure 2b. I do expect a domesticated species to show a U-shaped distribution of derived allele frequencies, but I have no similar expectation for a ratio of allele frequencies.*

**Response:** The ratio used in Fig. 2b was aimed to better show the comparison of allele frequency change for domestication targeted regions versus non-targeted regions. As cultivated rice has undergone domestication, the derived allele frequency (DAF) of domestication-targeted regions will shift to be much more fixed than non-targeted regions (as you can see for the *temperate japonica* group in orange, Fig. 2b). For wild rice (in green), as expected, no fixed pattern was observed. By applying this metric to weedy rice, we can demonstrate that weedy rice shows the same ratio pattern as cultivated rice, which indicates that domestication signatures remain in the genomes of the descendant weedy rice populations. The ratios as presented therefore serve as a useful illustration that weedy rice populations are derived primarily from domesticated rice ancestors.

*- L426-437 – What populations are these? Does this include all the weedy rice accessions?*
**Response:** As we hoped to examine whether weedy rice with the Ala205Val mutation show ALS-inhibiting herbicide resistance, the Italian weedy rice population (with one accession harboring the mutation) was selected to do this experiment. The experiment result is shown in Additional file 2: Fig. S9b.

*Results: 
- ALS herbicide resistance: In L169-173 you state that because some of the herbicide resistance mutations found in domesticated rice are also present in weedy rice this indicates introgression of the alleles from the crop to the weed. While this is the most parsimonious explanation, you have not eliminated the other possibility, which is parallel mutation through mutations at the same sites. This is not uncommon in the evolution of herbicide resistance, and must be addressed in the current manuscript.*

**Response:** We do agree with your comment that we could not eliminate the possibility that these herbicide resistance alleles are raised through parallel mutations in weedy populations. Therefore, for this revision, we have modified the conclusion of the paragraph as “These results suggest that HR weedy rice has likely acquired resistance by crop-weed hybridization and adaptive introgression — i.e., escape of resistance alleles from HR cultivars — although the possibility of parallel HR evolution in weedy rice by mutational convergence cannot be ruled out with the present data.”

*- L267: I can’t quite understand the meaning of “in the same cluster and separate with” as you describe the phylogeny here.*

**Response:** We meant that for the *GD1* gene, the *japonica* weedy rice group (colored in orange) and *japonica* cultivated rice (colored in brown) are almost completely separated into different clades, suggesting distinct haplotype of this gene between weedy and cultivated *japonica* type rice. We have modified the sentence as “The phylogeny of *GD1* clearly shows that most *japonica* weedy rice strains are separated from the group of *japonica* cultivated rice, which is consistent with the haplotype of this gene (Fig. 5c).”

- L 269: Did you mean “is different” instead of “is differed”?
**Response:** The sentence has been modified “In addition, we found one non-synonymous SNP on the last exon, and the allele frequency is markedly different between *japonica* weedy (0.85) and cultivated (0.12) rice.”

*Discussion:
- L277-279: Here, you say that “most weeds are derived from a single genetic background”. I assume you mean that most are not hybrids, but it sounds like you are saying that most are derived from the same founder population. I would suggest revising.*

**Response**: We have rephased the sentence as “most weeds are derived from a single genetic founder population (*indica*, *japonica*, or *aus*)”.

*- General suggestions for the origin of weeds from green revolution cultivars: I thought that this was perhaps the most interesting finding of the paper. However, I didn’t realize how many of the 524 weedy accessions had been derived from these lines until I looked at TableS5 – I believe the total is 269 (unless some weeds are double-counted)? If the total is really this high, please add it to the body of the paper – it will make a much more impressive impact if the reader can see that over 50% of weeds are derived from GR lines. Also, I think it might be pertinent to mention that this was a time when farming methods advanced considerably around the world. It is possible that a shift in farming methods contributed to the coordinated rise of these weeds.*
**Response**: We greatly appreciate your positive comments. we do also feel the origin of weeds from green revolution cultivars is the most interesting finding in this research. When tracing the ancestry by Kinship analysis with a panel of ~4,591 rice accessions as background, we found a high number of weedy rice samples have very high kinship value with some cultivars (green revolution cultivars or landraces) in very close geographic regions. As we could not find the detailed breeding history for related cultivars in some countries, we felt it is not easy to exactly determine whether the weed is derived from green revolution cultivars or landraces. Therefore, we selected a green revolution cultivar ‘Nanjing11’ with clear breeding history and known pedigrees, and confirmed the weedy rice group (Jiangsu: 22; Zhejiang: 4; Guangdong: 1) is likely to be derived from Nanjing11, not its parental lines before Green Revolution. Considering the number of sampled weeds of these three provinces in southern China is 75, we can estimate that more than 35% (27/75) of the current weedy rice strains in southern China (Jiangsu, Zhejiang, Guangdong) are likely descended from Green Revolution cultivars. We have added this estimated ratio in our revised version of manuscript.

For the possible reason for the rise of these weeds, we have taken your suggestion and added it in the result section: “These patterns suggest that a large proportion of Asian weed strains are descended from commercial cultivars that were widely grown in the 20th century, as rice agriculture shifted from smallholder farms to industrialized production.” Also, we mentioned it in the discussion “The proliferation of modern Asian weed strains has followed widespread shifts during the Green Revolution towards industrialized rice farming and adoption of a few high-yielding elite cultivars, which our data show to be direct founders of many contemporary Asian weed strains.”
